# Prognostic Value and Immune-Infiltration Pattern of *KIF4A* in Patients with Endometrial Carcinoma

**DOI:** 10.1155/2022/9621701

**Published:** 2022-01-17

**Authors:** Zhujuan Yang, Xiaoqing Shen, Shanhui Luo, Yi Li

**Affiliations:** ^1^Department of Gynecology, The Second Affiliated Hospital of Soochow University, Suzhou, China; ^2^Department of Gynecology, The Affiliated Jiangsu Shengze Hospital of Nanjing Medical University & Jiangsu Shengze Hospital, Suzhou, China

## Abstract

**Background:**

With the development of sequencing technology, an increasing number of biomarkers has been identified in endometrial carcinoma (EC). However, there have been few comprehensive analyses of the *KIF4A* gene in patients with EC.

**Methods:**

Based on raw data in public databases, the *KIF4A* gene and protein expression in EC were validated. Logistic regression analysis was conducted to analyze the correlations between clinical characteristics and the *KIF4A* expression. Kaplan-Meier analysis was used to explore the difference in survival in clinical subgroups. Meanwhile, we used meta-analysis in multiple datasets to investigate the prognostic value of *KIF4A*. In addition, Cox regression analysis was used to confirm the independent prognostic value of *KIF4A*, and we constructed a nomogram based on *KIF4A* expression. Subsequently, we used ESTIMATE and ssGSEA algorithms to excavate the correlation between *KIF4A*, tumour-infiltrating immune cells, and related gene markers of immune cells. Moreover, the potential biological functions of *KIF4A* were investigated by gene function annotation. Finally, we identified the hub genes interacting with *KIF4A* by constructing a protein-protein interaction (PPI) network and screening differential genes (DEGs).

**Results:**

In the pan-cancer analysis, *KIF4A* was upregulated in most tumors (21/33). Similarly, the overexpression of *KIF4A* in EC patients was confirmed in the TCGA cohort, the GEO cohort, and immunohistochemistry. In addition, upregulated *KIF4A* is associated with age, survival status, grade, FIGO stage, histological type, tumour invasion, and TCGA molecular subtypes (*p* < 0.05). *KIF4A* overexpression was correlated with the grade, histological type, and pathological stage according to logistic regression analysis (*p* < 0.05). Meanwhile, survival analysis and meta-analysis revealed that *KIF4A* was associated with a poor prognosis and acted as an independent prognostic marker in EC patients (*p* < 0.05). *KIF4A* is associated to immune response and may have a function in controlling immune cell infiltration in EC (20/24, *p* < 0.05). This is noteworthy given that gene enrichment analysis suggested KIF4A may be involved in the neuroactive ligand-receptor interaction pathway, etc. Finally, we identified transcription factors which have a potential interaction with *KIF4A*.

**Conclusion:**

We provided robust evidences that *KIF4A* is an indicator of poor prognosis and a potential target for immunotherapy in patients with EC.

## 1. Introduction

Endometrial cancer (EC) is a reproductive system malignancy that affects women's health, with over 200,000 new cases diagnosed each year worldwide. It is worth noting that EC patients are becoming younger [1]. In 2020, the National Comprehensive Cancer Network (NCCN) included TCGA molecular subtypes within its guidelines, and it is recommended for predicting prognosis and guiding treatment [2]. Therefore, it is necessary to explore further biomarkers of targeted therapies for EC based on the TCGA database.

*KIF4A* belongs to the kinesin superfamily, and its location and function in the nucleus change depending on the cell cycle stage [3]. *KIF4A* is closely related to the formation of a spindle during mitosis and the completion of cytoplasmic division [4] and intracellular transport of chemicals [5], because DNA damage can lead to abnormal cell proliferation and differentiation, which ultimately promotes tumour formation [6].

*KIF4A* is involved in the DNA damage repair pathway [6], so we speculate that *KIF4A* is closely related to the occurrence and prognosis of tumors. Numerous investigations on the biological process of *KIF4A* in ovarian cancer [7], cholangiocarcinoma [8], esophageal cancer [9], and bladder cancer [10] have been conducted. To our knowledge, only one study has focused on the role of *KIF4A* as the lncRNA LINC01123 target-gene in EC [11]. However, the intricate regulatory mechanism and prognostic value of KIF4A in EC is unclear.

In this study, we selected several public databases to validate the expression of KIF4A in EC patients. In the meantime, we used meta-analysis and Kaplan-Meier analysis to investigate its prognostic value in different clinical subgroups and demonstrated the independent prognostic value. We developed a visual prognostic model (nomogram) based on KIF4A expression. In addition, we explored the differences in immune infiltration between EC patients with different expression levels of KIF4A. Notably, the PPI network predicted a potential gene (transcription factor NFIB) interacting with KIF4A.

## 2. Materials and Methods

### 2.1. Datasets

Following the method of reference [12], we download RNA-sequence data (level-3 HTseq-FPKM) from the Pan-Cancer Project in The Cancer Genome Atlas (TCGA) database. In addition, the Uterine Corpus Endometrial Carcinoma (UCEC) RNA-sequence data was downloaded from the TCGA database to perform *KIF4A* difference analysis of paired (22 pairs) and unpaired (552 endometrial cancer tissue and 35 adjacent normal endometrial tissue). External validation of GSE17025 datasets (92 endometrial cancer tissue and 12 adjacent normal endometrial tissue) was downloaded from the GEO database. The expression of *KIF4A* protein was excavated from the Human Protein Atlas (HPA) database.

### 2.2. Prognosis Analysis

Clinical data were extracted from the TCGA database. FIGO stage, age, BMI, histological type, histologic grade, tumor invasion, and residual tumor are included in the clinical features; survival data include Overall Survival (OS), Disease-Specific Survival (DSS), and Progress-Free Interval (PFI), as reported in Table S1. We determined the median KIF4A expression in EC patients and utilized this value to create “high-KIF4A” and “low-KIF4A” groups. Kaplan-Meier survival analysis and the log-rank test indicate that there are differences in survival outcomes (OS, DSS, and PFI) between two groups. Additionally, univariate and multivariate Cox regression analyses were used to identify independent prognostic variables. Additionally, univariate and multivariate Cox regression analyses were used to identify independent prognostic variables. Additionally, we utilized the R software package “rms” to generate a nomogram to visualize the prognostic value of KIF4A. The ROC curve and calibration curve were used to examine the distinction and calibration.

### 2.3. Enrichment Analysis

The differentially expressed genes (DEGs) in *KIF4A*-high samples and *KIF4A*-low samples were screened using the “deseq2” package in R software. The thresholds were set to ∣log2(FC) | >2 and p.adj < 0.05. Moreover, Gene Ontology (GO) and Kyoto Encyclopedia of Genes and Genomes (KEGG) analyses were performed using related packages. The thresholds were set to p.adj < 0.05 and qvalue < 0.2.

### 2.4. Immune Analysis

The immune infiltration algorithm used in this study was ssGSEA. In addition, we used ESTIMATE algorithm to speculate the infiltration of stromal and immune cells in EC tissues from the TCGA database. We combined the above two algorithms to estimate the relationship between the expression of *KIF4A* and immune infiltration, including immune score, stromal score, ESTIMATE score, and immune cell correlation.

### 2.5. Construction of PPI Network

In order to screen the potentially interacting molecules with *KIF4A* in EC patients, the PPI network was constructed by using STRING and Cytoscape software. We retained the top 50 genes with the interaction relationship with *KIF4A* for the construction of the PPI network.

### 2.6. Statistical Analysis

All statistical analyses were performed using the R software (v.3.6.3). Detailed statistical methods are covered in the bioinformatics method section. *p* < 0.05 was considered statistically significant.

## 3. Result

### 3.1. *KIF4A* Expression in Pan-Cancer and EC Patients

**Figure 1:**
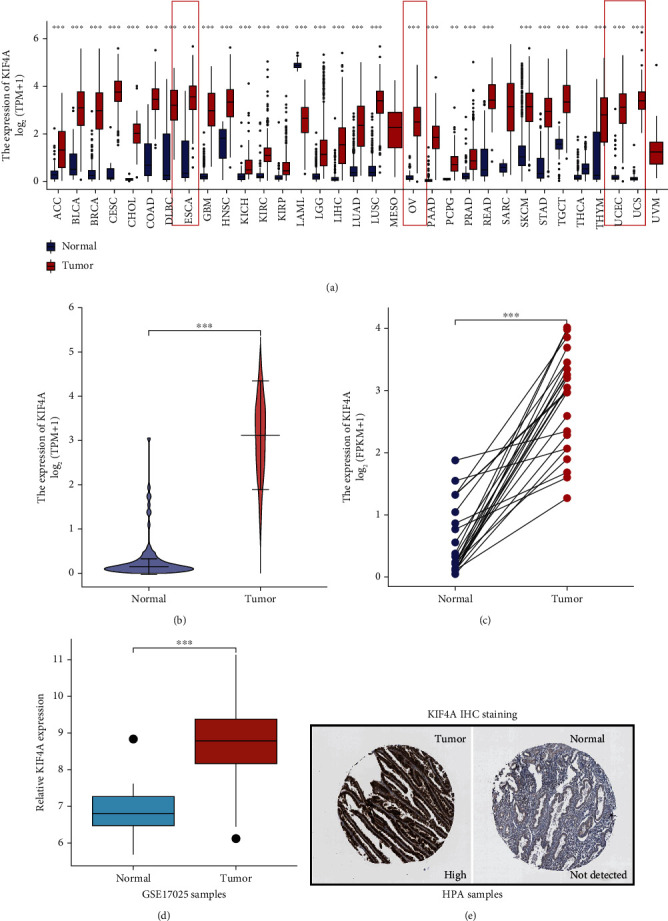
*KIF4A* expression in pan-cancers and EC patients. (a) Differential expression of *KIF4A* in pan-caner patients; gynecological tumors in the red frames. (b) The expression of *KIF4A* was explored by combining normal tissue samples in the GTEx database. (c) Differential expression *KIF4A* of paired samples from the TCGA database. (d) Differential expression *KIF4A* in GSE17025. (e) Representative immunohistochemical staining of *KIF4A* in the HPA database. ^∗∗^*p* < 0.01, ^∗∗∗^*p* < 0.001.

Firstly, we identified the expression of *KIF4A* in various cancers and focused on EC. We found that there were significant differences in the expression of *KIF4A* in pan-caner patients (21/33, *p* < 0.05), as shown in Figure 1(a). Of note, *KIF4A* was upregulated in all gynecological tumors. In addition, we explored expression of *KIF4A* by combining normal endometrial tissue samples in the GTEx database, and the same results were found (*p* < 0.001, Figure 1(b)). Meanwhile, significant upregulation of *KIF4A* was also observed in paired samples (Figure 1(c)) and the GSE17025 cohort (Figure 1(d)). Not surprisingly, representative Immunohistochemical staining of *KIF4A* protein in the HPA database revealed a lower expression in normal samples (Figure 1(e)).

### 3.2. Identification of the Correlation between *KIF4A* Expression and Clinical Characteristics

**Figure 2:**
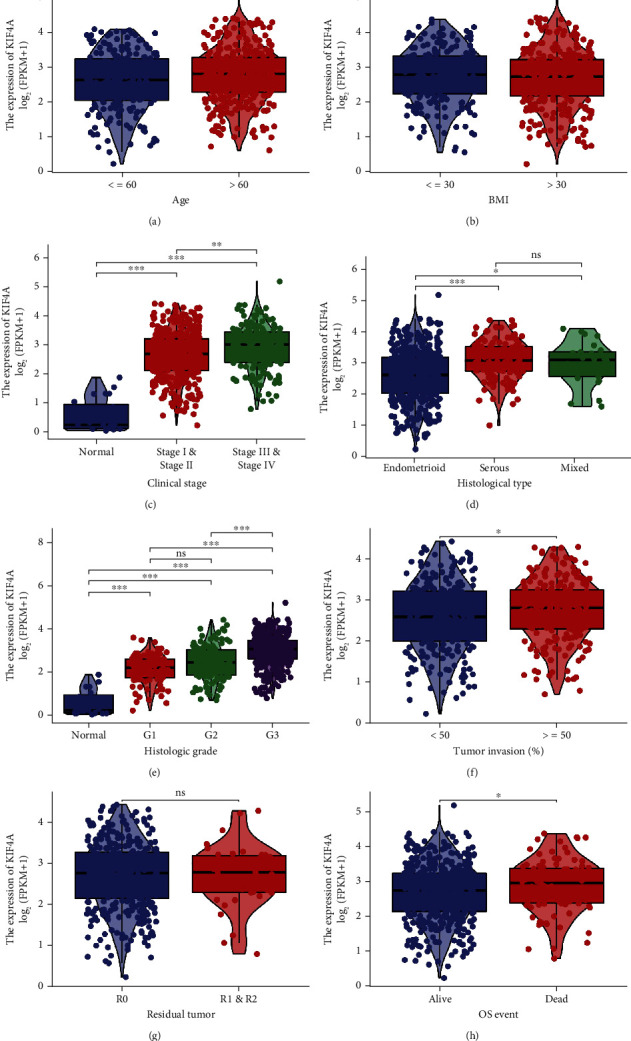
Identification of the correlation between *KIF4A* expression and clinical characteristics: (a) age, (b) BMI, (c) FIGO stage, (d) histological type, (e) histologic grade, (f) tumor invasion, (g) tumor residual, and (h) OS event. ^∗^*p* < 0.05, ^∗∗^*p* < 0.01, and ^∗∗∗^*p* < 0.001.

Correlation analysis was performed between the *KIF4A* expression and corresponding clinical characteristics. As presented in Figure 2, the increased expression of *KIF4A* is remarkably related to multiple factors, including age (Figure 2(a)), FIGO stage (Figure 2(c)), histological type (Figure 2(d)), grade (Figure 2(e)), tumor invasion (Figure 2(f)), and OS status (Figure 2(h)) (*p* < 0.05). Meanwhile, it should also be noted that the expression of *KIF4A* was not statistically correlated with the following clinical characteristics: BMI (Figure 2(b)) and tumor residual (Figure 2(h)). Furthermore, as shown in Table 1, using the median of *KIF4A* expression as the dependent variable, logistic regression analysis revealed that overexpression *KIF4A* was significant with the FIGO stage (advanced stage vs. early stage, *p* < 0.001), grade (G3 vs. G1 and G2, *p* < 0.001) and histological type (mixed and serous vs. endometrioid, *p* < 0.001).

### 3.3. Prognostic Value of *KIF4A* in EC Patients

**Figure 3:**
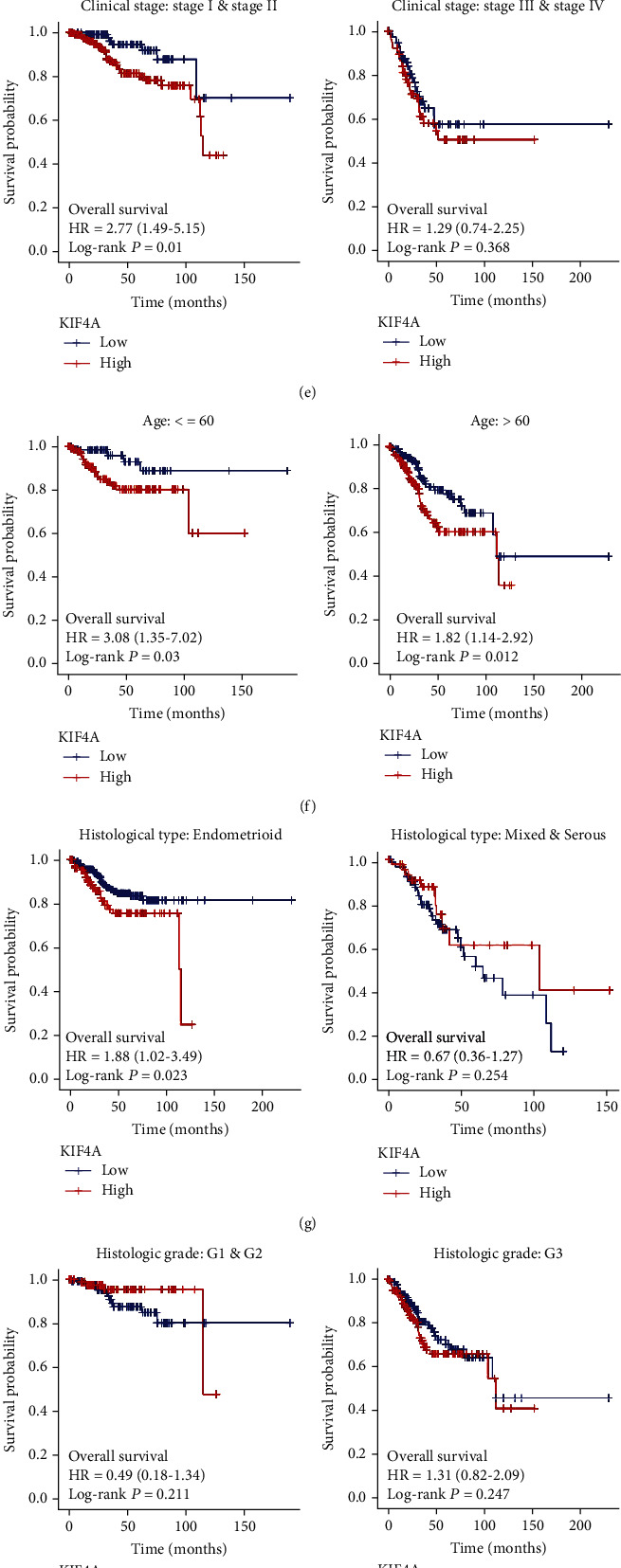
Prognostic value of *KIF4A* in EC Patients. Kaplan-Meier survival analysis of (a) OS, (b) DSS, (c) PFI, and (d) ROC analysis for status (tumor or normal). Survival analysis of clinical subgroups, including (e) FIGO stage, (f) age, (g) histological type, (h) histologic grade, (i) venous invasion, and (j) BMI.

To further explore the prognostic value of *KIF4A*, we performed survival analysis of the clinical subgroup. Firstly, we calculated the median expression of *KIF4A* of EC patients, which is used to select “high-*KIF4A*” and “low-*KIF4A*” groups. Kaplan-Meier survival analysis and log-rank test were used to suggest the survival differences (OS, DSS, and PFI) in two groups. The results showed that the survival time of the high-expression group was significantly shorter than that of the low-expression group (OS, *p* = 0.0003, Figure 3(a); DFS, *p* = 0.006, Figure 3(b); PFI, *p* < 0.001, Figure 3(c)). In addition, we also analyzed the prediction value of *KIF4A* in status (tumor or normal), and the ROC curve results showed that *KIF4A* had a terrific predictive performance (AUC:0.978, Figure 3(d)). In the survival analysis of the clinical subgroups, the survival time of the high-*KIF4A* group was significantly shorter than that of the low-*KIF4A* group in the early group (*p* = 0.01, Figure 3(e)), elderly and nonelderly group (*p* = 0.012/0.03, Figure 3(f)), endometrioid group (*p* = 0.023, Figure 3(g)), tumor invasion (%) < 50 group (*p* = 0.009, Figure 3(i)), and BMI > 30 group (*p* = 0.012, Figure 3(j)). Although there was no significant difference in survival time among the subgroups of the advanced stage group, mixed and serous group, grade subgroups (Figure 3(h)), tumor invasion (%) ≥ 50 group, and BMI < 30 group, it is of concern.

### 3.4. Meta-Analysis of the Prognostic Value and Comparison of TCGA Molecular Subtypes

**Figure 4:**
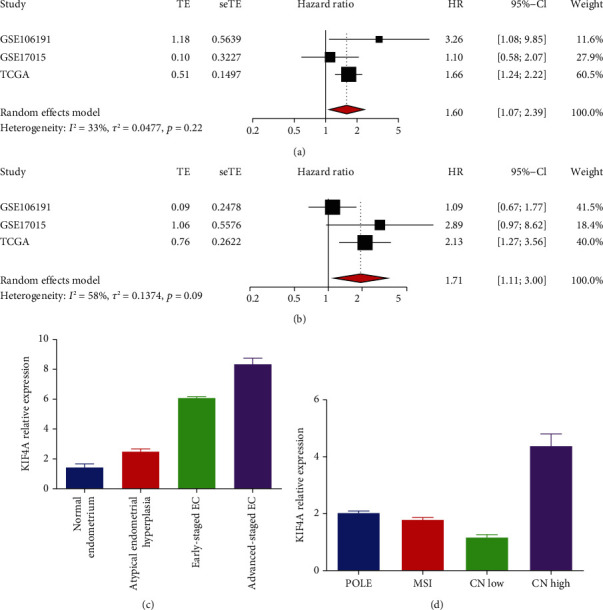
Meta-analysis of the prognostic value and comparison of TCGA molecular subtypes. (a) Meta-analysis of OS in EC patients. (b) Meta-analysis of PFS in EC patients. The relationship between *KIF4A* expression and degree of the lesion (c) and TCGA molecular subtypes (d).

To further illustrate the prognostic value of *KIF4A* and correlation with TCGA molecular subgroups, we performed meta-analysis of *KIF4A* by combining three datasets (TCGA, GSE106191, and GSE17015), where we used the random effects model. Among the results in the meta-analysis of OS, *KIF4A* was shown to be a high risk factor for survival in EC patients, HR = 1.60 (1.07-23.9), as shown in Figure 4(a). Meanwhile, the same results were shown in the meta-analysis of PFS, HR = 1.71 (1.11-3.00), as shown in Figure 4(b). We analyzed the correlation between KIF4A expression and cancer progression, and TCGA molecular subtypes in the GEO dataset. With the progression of tumor, the expression of *KIF4A* was upregulated (Figure 4(c), *p* < 0.05). In addition, it is interesting to note that *KIF4A* expression is highest in CN-high (Figure 4(d), *p* < 0.05).

### 3.5. Construction of Nomogram Based on *KIF4A*

**Figure 5:**
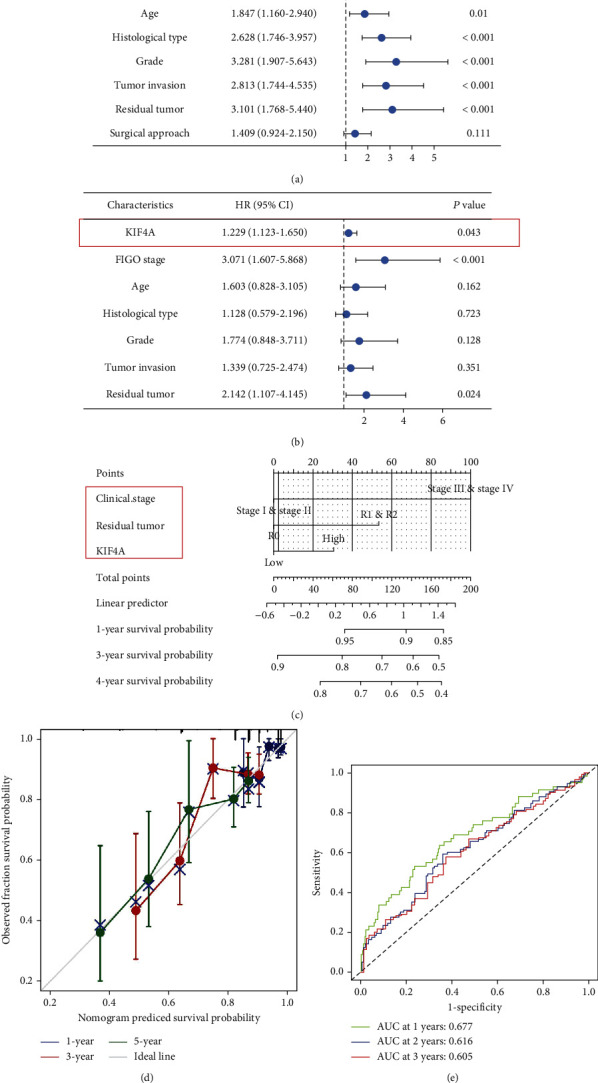
Construction of nomogram based on *KIF4A*. Univariate (a) and multivariate Cox regression analyses (b) based on *KIF4A* and clinicopathologic factors: nomogram (c), calibration curve (d), and ROC analysis (e).

The independent prognostic value of *KIF4A* in EC patients was investigated, in which univariate Cox analysis revealed that *KIF4A* was a high-risk factor (Figure 5(a)). Moreover, further multivariate Cox analysis showed that *KIF4A*, FIGO stage, and tumor residual were independently associated with OS, which may imply that *KIF4A* may be an independent prognostic predictor for EC patients (Figure 5(b)). Meanwhile, we combined significance factors in multivariate analysis to construct a visual prognostic model (Figure 5(c)). The ROC curve and calibration curve also showed that the model had better predictive value (Figures 5(d) and 5(e)).

### 3.6. Correlation between *KIF4A* Expression and Immune Infiltration in EC

**Figure 6:**
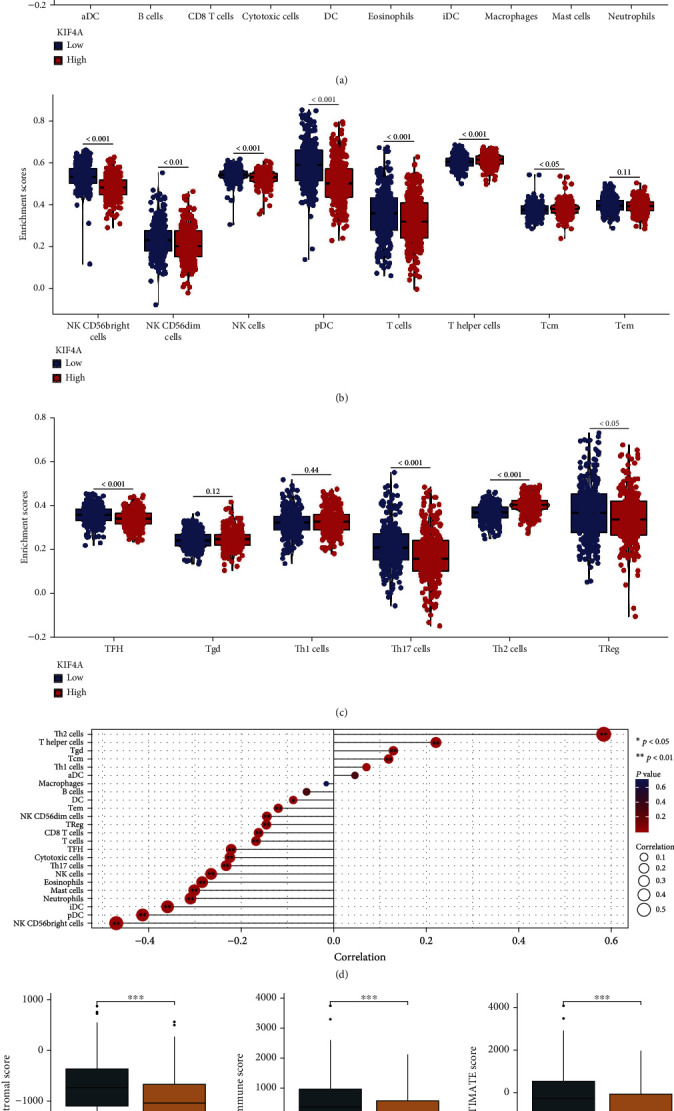
Correlation between *KIF4A* expression and immune infiltration in EC. (a–c) Differential expression analysis of 24 immune cells in patients with different *KIF4A* expressions. (d) Pearson analysis of 24 immune cells and *CRIP1* expression; comparison of stromal scores (e), immune scores (f), and ESTIMATE scores (g) based on the ESTIMATE tool. ^∗^*p* < 0.05, ^∗∗^*p* < 0.01, ^∗∗∗^*p* < 0.001.

In particular, infiltrating immune cells are an independent predictor of survival in patients with EC. Therefore, we explored the correlation between *KIF4A* and 24 immune cells, as well as the relationship between the expression of *KIF4A* mRNA and immune cells using the ssGSEA algorithm. Based on the median expression value of *KIF4A*, all EC patients were classified into high- and low-expression groups. The results showed that most of the cells were significantly different between groups except for aDC, B cell, DC, macrophages, Tem, Tgd, and Th1 cells (Figures 6(a)–6(c), *p* < 0.05). Meanwhile, the results showed that *KIF4A* was correlated with most immune cells, except Th1 cells, aDC, macrophages, and B cells (Figure 6(d)). In addition, ESTIMATE analysis revealed that the low-*KIF4A* group had a higher immune and stromal score than the high-*KIF4A* group (Figures 6(e)–6(g)). Finally, Pearson analysis showed that KIF4A expression correlated with the expression of most immune cell marker genes (Table 2, *p* < 0.05).

### 3.7. Analysis of the Potential Mechanisms of *KIF4A*

**Figure 7:**
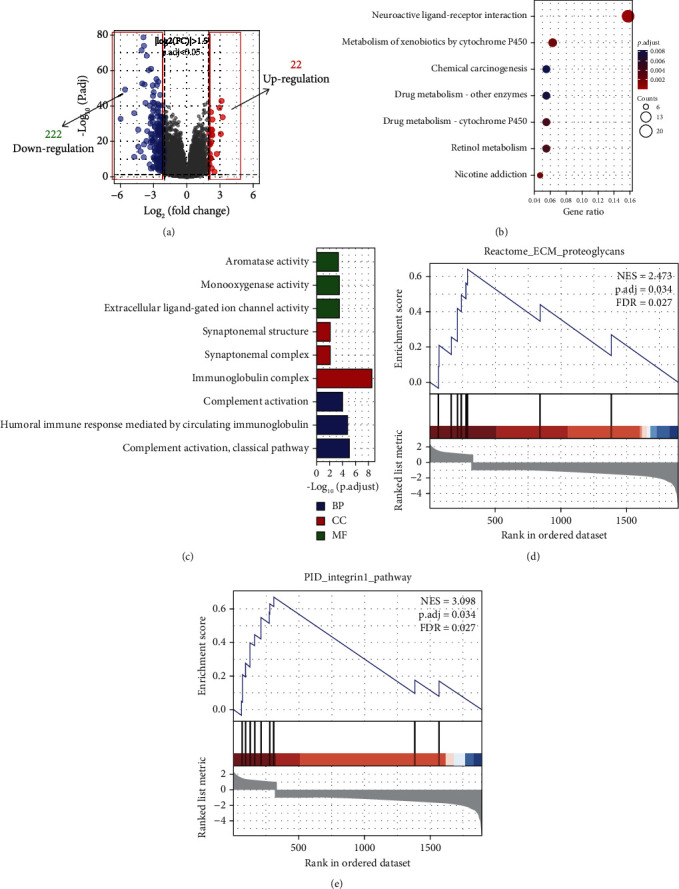
Analysis of the potential mechanisms of *KIF4A*. (a) Volcano map of DEGs. (b) GO enrichment analysis. (c) KEGG enrichment analysis.(d, e) GSEA enrichment analysis.

In order to explore the potential mechanism of *KIF4A* involvement, we performed gene enrichment analysis on all the DEGs from high- and low-risk groups (Figure 7(a)). Based on the results of the KEGG pathway analysis of DEGs, the genes were mainly enriched in neuroactive ligand-receptor interaction, metabolism of xenobiotics by cytochrome P450, etc. (Figure 7(b)**)**. Based on the results of the GO enrichment analysis of DEGs, the genes were mainly enriched in complement activation, classical pathway, humoral immune response mediated by circulating immunoglobulin, complement activation, etc. (Figure 7(c)**)**. In addition, we performed GSEA enrichment analysis of the above genes, and we show some of the results in Figures 7(d) and 7(e) and others in Table S2. GSEA showed that these genes may be associated with biological oxidations, etc.

### 3.8. A Prediction about Protein-Protein Interaction of *KIF4A*

**Figure 8:**
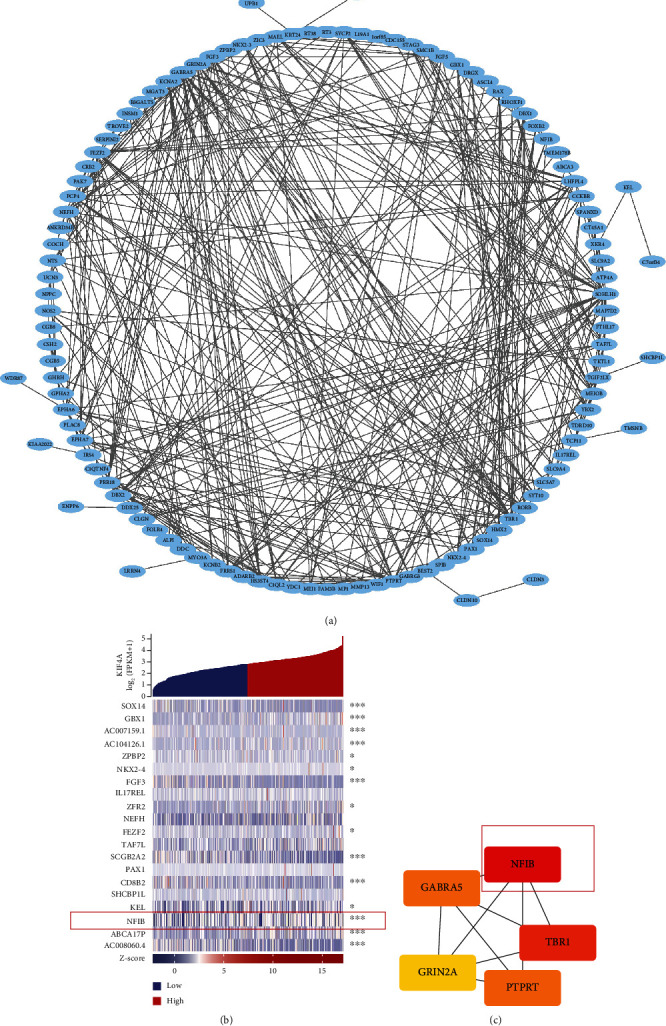
A prediction about protein-protein interaction of *KIF4A*: (a) PPI network map of DEGs; (b) a heat map of top 30 DEGs; (c) hub genes in PPI network.

To identify potential interaction networks in the above 244 DEGs, we constructed a circular PPI network based on the STRING database and Cytoscape software (Figure 8(a)). Meanwhile, the heat map showed the top 20 genes of |logFC|, and the correlation with *KIF4A* was shown on the right side of the heat map (Figure 8(b)). In addition, we performed topological analysis of the genes in the PPI network by Cytoscape and, finally, identified five hub genes (Figure 8(c)). Finally, we identified *NFIB*, which may be closely related to *KIF4A* (*p* < 0.001). Given that *NFIB* is a transcription factor, we speculated that the promoter of *KIF4A* has a potential *NFIB* site; however, this needs to be verified by future experiments.

## 4. Discussion

We conducted a detailed examination of *KIF4A* in EC in this study. We analyzed *KIF4A*'s genetic landscape across several datasets and discovered a strong association between *KIF4A* and clinicopathological variables. Simultaneously, several investigations established *KIF4A*'s remarkable prognostic potential. Additionally, we investigated the possible mechanism of *KIF4A* and its effect on immunological function. Finally, based on 244 DEGs, NFBI was the most critical transcription factor controlled upstream of *KIF4A*. However, to the best of our knowledge, the role of *KIF4A* in EC remains unknown.

Because of its adverse consequences, EC has gotten a lot of attention [2]. In recent decades, the global incidence has risen [1]. Immunotherapy has shown some promise in the treatment of a variety of cancers in recent research [13]. As a result, biomarkers discovered in this work, *KIF4A* can be utilized to assess treatment responsiveness and survival outcomes in EC patients. In addition, PPI networks have been extensively investigated, and its topology is frequently linked to diseases [14, 15]. Therefore, NFIB, a hubl gene found in our study, may interact with *KIF4A*.

This study has several limitations. Firstly, our survival and *KIF4A* expression analyses were derived from public databases, mainly Caucasians. Therefore, the lack of clinical information from other ethnic groups may result in poor extrapolation. Second, no laboratory experiments were performed to validate the mechanism of *KIF4A* with NFIB. This also indicates that the role of *KIF4A* in EC is the focus of our future research. Finally, since the TCGA-EC cohort had relatively fewer stage III-IV samples than stage I-II, our conclusion may be more accurate in predicting early stage EC patients.

## 5. Conclusions

This study has identified some novel DEGs and pathways in EC. More specifically, *KIF4A* is a hub gene with the ability to promote EC progression and affect the immune microenvironment. We provide a potential transcription factor (NFIB) associated with *KIF4A* that can be used in subsequent studies. Our findings provide researchers with potential therapeutic targets for EC therapy.

## Figures and Tables

**Table 1:** Logistic regression analysis between *KIF4A* expression and clinical characteristics.

Characteristics	Odds ratio (OR)	*p* value
Age (>60 vs. ≤60)	1.358 (0.960-1.922)	0.084
Grade (G3 vs. G1 & G2)	6.373 (4.354-9.448)	<0.001
Histological type (mixed & serous vs. endometrioid)	2.903 (1.946-4.387)	<0.001
FIGO stage (stage III & stage IV vs. stage I & stage II)	1.941 (1.335-2.837)	<0.001
Tumor invasion (%) (≥50 vs. <50)	1.401 (0.975-2.017)	0.069

**Table 2:** Correlation analysis between KIF4A and related gene markers of immune cells.

Immune cells	Gene marker	*R* value	*p* value
CD8+ T cell	CD8A	-0.044	0.305
	CD8B	-0.168	<0.001

T cell (general)	CD3D	-0.193	<0.001
	CD3E	-0.190	<0.001
	CD2	-0.124	0.004

B cell	CD19	-0.010	0.814
	CD79A	-0.089	0.037

Monocyte	CD86	-0.021	0.622
	CSF1R	-0.227	<0.001

TAM	CCL2	-0.104	0.015
	CD68	0.116	0.006
	IL10	-0.045	0.289

M1 macrophage	NOS2	-0.040	0.344
	IRF5	-0.007	0.868
	PTGS2	-0.094	0.027

M2 macrophage	CD163	0.142	<0.001
	VSIG4	0.034	0.426
	MS4A4A	0.012	0.770

Neutrophils	CEACAM8	-0.029	0.498
	ITGAM	-0.137	0.001
	CCR7	-0.170	<0.001

Natural killer cell	KIR2DL1	-0.041	0.340
	KIR2DL3	-0.041	0.334
	KIR2DL4	-0.044	0.300
	KIR3DL1	-0.019	0.660
	KIR3DL2	-0.001	0.988
	KIR3DL3	-0.049	0.251
	KIR2DS4	-0.034	0.423

Dendritic cell	HLA-DPB1	-0.257	<0.001
	HLA-DQB1	-0.288	<0.001
	HLA-DRA	-0.236	<0.001
	HLA-DPA1	-0.231	<0.001
	CD1C	-0.267	<0.001
	NRP1	0.004	0.916
	ITGAX	-0.198	<0.001

Th1 cell	TBX21	-0.060	0.159
	STAT4	-0.111	0.009
	STAT1	0.394	<0.001
	IFNG	0.056	0.189
	TNF	0.080	0.060

Th2 cell	GATA3	-0.060	0.162
	STAT6	-0.235	<0.001
	STAT5A	-0.127	0.003
	IL13	-0.060	0.160

Tfh cell	BCL6	-0.264	<0.001
	IL21	0.086	0.043

Th17 cell	STAT3	-0.048	0.255
	IL17A	0.016	0.701

Treg cell	FOXP3	-0.101	0.018
	CCR8	0.090	0.034
	STAT5B	-0.065	0.128
	TGFB1	-0.208	<0.001

T cell exhaustion	PDCD1	-0.138	0.001
	CTLA4	-0.148	<0.001
	LAG3	0.062	0.145
	HAVCR2	-0.026	0.545
	GZMB	-0.022	0.600

## Data Availability

The following information was supplied regarding data availability: data is available at the TCGA database (https://portal.gdc.cancer.gov/).

## Supplementary Materials

Supplementary 1 Table S1: *KIF4A* expression and clinical characteristics of EC patients in TCGA database (excluded unknown samples).

Supplementary 2 Table S2: results of GSEA analysis based on high- and low-risk groups.
