# Supplementary material for: Prognostic Value and Immune-Infiltration Pattern of KIF4A in Patients with Endometrial Carcinoma
Source: Dis Markers. 2022 Jan 17;2022:9621701. doi: 10.1155/2022/9621701 (PMC8814714; doi:10.1155/2022/9621701)
Supplement: Supplementary 1 — Table S1: KIF4A expression and clinical characteristics of EC patients in TCGA database (excluded unknown samples). [file 9621701.f1.docx]

| Characteristic | levels | Low-*KIF4A* | High-*KIF4A* | p |
| --- | --- | --- | --- | --- |
| FIGO stage, n (%) | Stage I | 195 (35.3%) | 147 (26.6%) | < 0.001 |
|  | Stage II | 20 (3.6%) | 31 (5.6%) |  |
|  | Stage III | 49 (8.9%) | 81 (14.7%) |  |
|  | Stage IV | 12 (2.2%) | 17 (3.1%) |  |
| Age, n (%) | <=60 | 113 (20.6%) | 93 (16.9%) | 0.101 |
|  | >60 | 162 (29.5%) | 181 (33%) |  |
| BMI, n (%) | <=30 | 103 (19.8%) | 109 (21%) | 0.529 |
|  | >30 | 159 (30.6%) | 148 (28.5%) |  |
| Histological type, n (%) | Endometrioid | 232 (42%) | 178 (32.2%) | < 0.001 |
|  | Mixed | 8 (1.4%) | 16 (2.9%) |  |
|  | Serous | 36 (6.5%) | 82 (14.9%) |  |
| Histologic grade, n (%) | G1 | 83 (15.3%) | 15 (2.8%) | < 0.001 |
|  | G2 | 82 (15.2%) | 38 (7%) |  |
|  | G3 | 106 (19.6%) | 217 (40.1%) |  |
| Tumor invasion(%), n (%) | <50 | 147 (31%) | 112 (23.6%) | 0.084 |
|  | >=50 | 104 (21.9%) | 111 (23.4%) |  |
| Residual tumor, n (%) | R0 | 193 (46.7%) | 182 (44.1%) | 0.794 |
|  | R1 | 12 (2.9%) | 10 (2.4%) |  |
|  | R2 | 7 (1.7%) | 9 (2.2%) |  |

**TableS1. *KIF4A* expression and clinical characteristics of EC patients in TCGA database.**
