# Supplementary material for: Prognostic Value and Immune-Infiltration Pattern of KIF4A in Patients with Endometrial Carcinoma
Source: Dis Markers. 2022 Jan 17;2022:9621701. doi: 10.1155/2022/9621701 (PMC8814714; doi:10.1155/2022/9621701)
Supplement: Supplementary 2 — Table S2: results of GSEA analysis based on high- and low-risk groups. [file 9621701.f2.docx]

**TableS2. Results of GSEA analysis based on high - and low-risk groups**

| Description | setSize | enrichmentScore | NES | p.adjust | qvalues |
| --- | --- | --- | --- | --- | --- |
| REACTOME_BIOLOGICAL_OXIDATIONS | 32 | -0.434595008 | -2.162256154 | 0.034415584 | 0.026885395 |
| WP_METAPATHWAY_BIOTRANSFORMATION_PHASE_I_AND_II | 29 | -0.424873112 | -2.038022686 | 0.034415584 | 0.026885395 |
| KEGG_METABOLISM_OF_XENOBIOTICS_BY_CYTOCHROME_P450 | 11 | -0.615682131 | -2.099985143 | 0.034415584 | 0.026885395 |
| REACTOME_PHASE_I_FUNCTIONALIZATION_OF_COMPOUNDS | 20 | -0.470788587 | -2.007735712 | 0.034415584 | 0.026885395 |
| REACTOME_ECM_PROTEOGLYCANS | 10 | 0.638376885 | 2.472600358 | 0.034415584 | 0.026885395 |
| REACTOME_NON_INTEGRIN_MEMBRANE_ECM_INTERACTIONS | 10 | 0.651061744 | 2.521732132 | 0.034415584 | 0.026885395 |
| REACTOME_RESPONSE_TO_ELEVATED_PLATELET_CYTOSOLIC_CA2_ | 10 | 0.69475851 | 2.690981115 | 0.034415584 | 0.026885395 |
| WP_VEGFAVEGFR2_SIGNALING_PATHWAY | 10 | 0.654376148 | 2.534569683 | 0.034415584 | 0.026885395 |
| KEGG_DILATED_CARDIOMYOPATHY | 14 | 0.563696638 | 2.603068997 | 0.034415584 | 0.026885395 |
| KEGG_HYPERTROPHIC_CARDIOMYOPATHY_HCM | 14 | 0.644413259 | 2.975806599 | 0.034415584 | 0.026885395 |
| PID_INTEGRIN1_PATHWAY | 14 | 0.670804489 | 3.097677456 | 0.034415584 | 0.026885395 |
| REACTOME_STRIATED_MUSCLE_CONTRACTION | 13 | 0.840510367 | 3.734645969 | 0.034415584 | 0.026885395 |
| WP_STRIATED_MUSCLE_CONTRACTION_PATHWAY | 13 | 0.840510367 | 3.734645969 | 0.034415584 | 0.026885395 |
| KEGG_ECM_RECEPTOR_INTERACTION | 12 | 0.633463719 | 2.694126596 | 0.034415584 | 0.026885395 |
| WP_CARDIAC_PROGENITOR_DIFFERENTIATION | 12 | 0.526639981 | 2.239804327 | 0.034415584 | 0.026885395 |
| REACTOME_DEGRADATION_OF_THE_EXTRACELLULAR_MATRIX | 18 | 0.509938341 | 2.663771111 | 0.034415584 | 0.026885395 |
| KEGG_FOCAL_ADHESION | 21 | 0.571333356 | 3.251761213 | 0.034415584 | 0.026885395 |
| REACTOME_MUSCLE_CONTRACTION | 21 | 0.464550755 | 2.644004787 | 0.034415584 | 0.026885395 |
| WP_FOCAL_ADHESION | 20 | 0.644417854 | 3.579024737 | 0.034415584 | 0.026885395 |
| NABA_ECM_REGULATORS | 26 | 0.332578135 | 2.145964449 | 0.035546613 | 0.027768953 |
| REACTOME_EXTRACELLULAR_MATRIX_ORGANIZATION | 30 | 0.466281798 | 3.159885079 | 0.039278656 | 0.030684419 |
| NABA_CORE_MATRISOME | 35 | 0.317094792 | 2.313413746 | 0.041471049 | 0.032397112 |
| REACTOME_SIGNALING_BY_RECEPTOR_TYROSINE_KINASES | 36 | 0.275851079 | 2.008933905 | 0.041471049 | 0.032397112 |
| REACTOME_CYTOCHROME_P450_ARRANGED_BY_SUBSTRATE_TYPE | 16 | -0.501465888 | -1.956705278 | 0.041471049 | 0.032397112 |
| WP_OXIDATION_BY_CYTOCHROME_P450 | 16 | -0.502399528 | -1.960348317 | 0.041471049 | 0.032397112 |
| REACTOME_PLATELET_ACTIVATION_SIGNALING_AND_AGGREGATION | 13 | 0.478663801 | 2.126850431 | 0.041471049 | 0.032397112 |
